# Determination of the Lethal Concentrations of Two Phenolic Acid Derivatives Originated From the Edible Red Marine Macroalga (*Bangia fuscopurpurea*) Using the In Vivo Zebrafish Eleutheroembryo Model and Their In Silico Structure–Toxicity Relationship Study

**DOI:** 10.1002/fsn3.71182

**Published:** 2026-01-21

**Authors:** Shi‐Ying Huang, Guiling Li, Yi‐Jia Shih, Chang‐Wei Hsieh, Yun‐Sheng Lin, Jingwen Liu, Tao Sun, Chien‐Wei Feng

**Affiliations:** ^1^ College of Ocean Food and Biological Engineering Jimei University Xiamen China; ^2^ Fujian Marine Functional Food Engineering Technology Research Center Xiamen China; ^3^ Sustainable Ocean Governance Center National Sun Yat‐Sen University Kaohsiung Taiwan; ^4^ Department of Food Science and Biotechnology National Chung Hsing University Taichung Taiwan; ^5^ Department of Food Science National Ilan University Yilan Taiwan; ^6^ Department of Medical Research China Medical University Hospital Taichung Taiwan; ^7^ Department of Food Science National Pingtung University of Science and Technology Pingtung Taiwan; ^8^ Center for Precision Medicine Huaqiao University Xiamen China; ^9^ School of Medicine and School of Biomedical Sciences Huaqiao University Xiamen China; ^10^ Department of Obstetrics and Gynecology Kaohsiung Medical University Hospital, Kaohsiung Medical University Kaohsiung Taiwan; ^11^ Center for Cancer Research Kaohsiung Medical University Kaohsiung Taiwan

**Keywords:** in silico evaluation, in vivo lethal dose, phenolic acid derivatives, structure–toxicity relationship, zebrafish

## Abstract

A 2023 study identified two phenolic acid derivatives (HBP2–3) in the extract from the edible macroalga (*Bangia fuscopurpurea*), and we previously demonstrated the in vitro neuroprotective effects of HBP2–3. However, the appropriate starting experimental concentration range for HBP2–3 in animals remained unclear, and it was uncertain which compound might carry a lower toxicity risk. This study assessed the in vivo lethal dose of HBP2–3 and analyzed their in silico toxicological profiles to support a structure–toxicity relationship (STR) analysis. We predicted their LD_50_ using the tools GUSAR and DL‐AOT, and determined their lethal concentrations (LC) using the in vivo zebrafish eleutheroembryo model. We predicted their toxicological properties using the tools (ADMETlab 3.0, TISBE, and embryoTox). An in vitro model was further selected to assess their toxicity. In in silico models, HBP2–3 showed potential to treat 12 parkinsonian syndromes, and HBP2 exhibited a higher rat oral LD_50_ than HBP3. In the in vivo zebrafish eleutheroembryo model, HBP2 (0.1–200 μM) and HBP3 (0.1–10 μM) did not induce mortality, and median LC (LC_50_) of HBP3 was estimated to be 115.48 μM. Compared with HBP3, HBP2 exhibited the following in silico advantages: (a) lower probabilities of nephrotoxicity and neurotoxicity; and (b) a reduced risk of developmental toxicity. In in vitro human neuronal IMR‐32 cells, HBP3 exhibited greater cytotoxicity, potentially associated with the downregulation of Bcl‐2 and the activation of caspase‐3. These advantages of HBP2 may be associated with an increased degree of hydroxylation.

## Graphical abstract

This study compared the predicted and experimental toxicity of two phenolic acid derivatives (HBP2 and HBP3) from the edible macroalga *Bangia fuscopurpurea*. HBP2 exhibited greater in vivo safety and lower predicted toxicity risks in silico, whereas HBP3 displayed higher in vitro cytotoxicity in IMR‐32 cells, potentially associated with the downregulation of Bcl‐2 and the activation of caspase‐3. These advantages of HBP2 may be associated with an increased degree of hydroxylation.

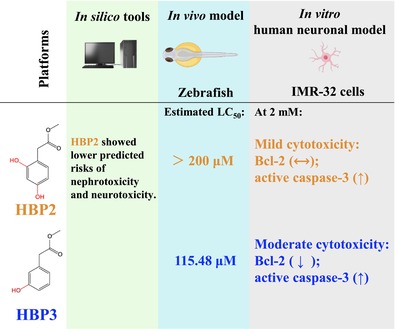

## 1 Introduction

### 1.1 Previous Studies on the Bioactivities of *Bangia fuscopurpurea*

The exploration of novel bioactivities of marine‐derived compounds and the structural analogues is a promising research area (Atanasov et al. 2021; Banerjee et al. 2022; Haque et al. 2022). Numerous research groups have focused on natural products from edible macroalgae (Cikoš et al. 2021), particularly red macroalgae (Aziz et al. 2020; Shih et al. 2017). The edible red macroalga *Bangia fuscopurpurea* has been one of “The Ten Outstanding Aquatic Germplasm Resources” designated by the Ministry of Agriculture and Rural Affairs of China in 2021 (Yan 2022). Besides its nutritional benefits, from a traditional perspective, it has also been believed to help reduce blood pressure and decrease the risk of vascular diseases (Zheng et al. 2002). However, database searches using the keyword “*Bangia fuscopurpurea*” (on June 25, 2025) resulted in only 27 articles in PubMed (using the “All fields” function) and 42 articles in Web of Science (using the “Topic” function), indicating considerable potential for further investigation into its bioactivities. Previous research on its bioactivities has primarily concentrated on two areas (Huang et al. 2024, 2020): (1) polysaccharides and (2) proteins, both derived from water extracts. Previous studies on its natural products have primarily focused on exploring new bioactivities, whereas only a few have addressed their toxicological properties. In the fields of food science and nutrition, toxicological research on natural products continues to receive sustained attention from scientists (Fatima et al. 2025; Li et al. 2025; Susu et al. 2025).

### 1.2 Focus of This Study: Two Phenolic Acid Derivatives (HBP2–3) Originated From *Bangia fuscopurpurea*

From 2020 to 2023, four research groups started to investigate the bioactivities of the ethanol extracts (Chang et al. 2023; Huang et al. 2020; Li et al. 2021) or its compound (phytol (Wang et al. 2023)) from *Bangia fuscopurpurea*. In 2023, two phenolic acid derivatives (HBP2–3) and a derivative were identified in its ethanol extract (Chang et al. 2023). We previously demonstrated the neuroprotective effects (for the in vitro model of Parkinson's disease) of the above three compounds in 2024, and further found that HBP2–3 had the highest therapeutic index (10,000) (Huang et al. 2024). However, for HBP2–3, it remains unclear which one may pose a relatively lower toxicity risk and thus greater development potential.

### 1.3 In Silico Assessment of the Compound's Toxicity Risks

It is crucial for a drug to have appropriate bioactivity and good pharmacokinetic properties, particularly low toxicity (Dulsat et al. 2023; Sun et al. 2022). The assessment of the median lethal dose (LD_50_) has been used as a major parameter in measuring acute toxicity, and also as an initial procedure for general screening of chemical and pharmacological agents for toxicity (Chinedu et al. 2013). Zebrafish exhibit a high degree of genetic and physiological homology with humans (Roohi et al. 2024). A review concluded that many genes encoding metabolic enzymes involved in drug detoxification in zebrafish have identifiable human orthologs (Cakan‐Akdogan et al. 2023). Zebrafish have been considered a successful in vivo animal model for toxicity screening of natural products (Chahardehi et al. 2020). For drug development, especially during dose–toxicity testing in animal models, compounds isolated from natural products require a continuous and cost‐feasible supply of sufficient quantities for evaluation (Atanasov et al. 2021), especially marine‐derived compounds (Huang et al. 2012). HBP2–3 already have available commercial product forms with purity equal to or greater than 98% (Huang et al. 2024). On the other hand, in the early phase of drug development, toxicity risk prediction could enhance the success probability of selecting compounds for lead optimization (Dulsat et al. 2023). In this study, we also utilized artificial intelligence (AI)‐based in silico tools to predict and compare the toxicity risks of HBP2–3.

### 1.4 The Experimental Design of This Study

In this study, we evaluated the in vivo lethal dose of HBP2–3 and performed an in silico comparison of their toxicological properties for a structure–toxicity relationship (STR) analysis. With the in silico tool DIGEP‐Pred 2.0, we examined the potential of HBP2–3 for treating parkinsonian syndromes. We predicted their in vivo LD_50_ using the in silico tools GUSAR and DL‐AOT. The in vivo toxicity assessments can be performed using zebrafish eleutheroembryos for estimating the biological safety of natural products derived from foods (Demirtas et al. 2025). Previous studies assessed the acute toxicity of either polyphenol‐rich extracts or synthetic phenolic antioxidants using zebrafish eleutheroembryos (Fernezelian et al. 2023; Yang et al. 2018). We determined the lethal concentration (LC) of HBP2–3 using the in vivo zebrafish eleutheroembryo model. We predicted their toxicological properties using the three in silico tools, including ADMETlab 3.0, TISBE, and embryoTox. The in silico tool DIGEP‐Pred 2.0 was utilized to explore their potential effects at the protein level. According to these in silico results, an in vitro human cell line model was subsequently selected to assess their possible toxicity and validate the predicted protein‐level effects. Based on the combined findings from in silico, in vivo, and in vitro analyses, this study suggests the test compound with a higher LC_50_ in the zebrafish eleutheroembryo model and lower predicted toxicity risks may possess greater potential for further development.

## 2 Methods

### 2.1 Test Compounds

HBP2: methyl 2,4‐dihydroxyphenylacetate or methyl 2‐(2,4‐dihydroxyphenyl)acetate (CAS: 67828‐42‐6; SMILES: COC(=O)CC1=C(C=C(C=C1)O)O; catalog no. HY‐W440039, 98%, MedChemExpress, New Jersey, USA).

HBP3: methyl 2‐(3‐hydroxyphenyl)acetate, methyl 3‐hydroxyphenylacetate, or 3‐hydroxyphenylacetic acid methyl ester (CAS: 42058‐59‐3; SMILES: COC (=O)CC1=CC(=CC=C1)O; catalog no. M2925, > 98%, Tokyo Chemical Industry, Tokyo, Japan).

### 2.2 Database Searches for Possible Related Publications About HBP2–3

We conducted literature searches for the numbers of possible related articles about HBP2–3 using three databases on June 25, 2025. Specifically, we searched the PubMed database (National Center for Biotechnology Information [NCBI], National Library of Medicine, Maryland, USA) (Lu 2011) by entering the chemical names of the test compounds in the “All Fields” option. For the Web of Science database (Web of Science Group, London, UK) (Birkle et al. 2020), we used the “Topic” field to perform keyword‐based searches with the chemical names. We retrieved literature information from the PubChem Compound (PubChem) database (NCBI) (Kim et al. 2016) by referring to the “Consolidated References” listed under the “Literature” section for each test compound.

### 2.3 In Silico Predictions of the Potential Effects of HBP2–3 on Parkinsonian Syndromes

The DIGEP‐Pred 2.0 tool (Ivanov et al. 2024) (Laboratory for Structure–Function Based Drug Design, Department of Bioinformatics, Institute of Biomedical Chemistry, Moscow, Russia), was used to predict the potential effects of HBP2–3 on gene expression profiles, based on protein expression data from the Comparative Toxicogenomics Database (CTD). Structure–activity relationship (SAR) analysis and biological activity prediction were performed using the Prediction of Activity Spectra for Substances (PASS) platform. PASS provides two indices: the “probability of being active (*Pa*)”, which estimates the likelihood that a compound belongs to the category of active substances (structurally similar to known active molecules), and the “probability of being inactive (*Pi*)”, which estimates the likelihood of inactivity based on structural similarity to known inactive compounds. Only predictions with *Pa*>*Pi* were considered indicative of possible activity. Gene enrichment analysis was then conducted for those genes predicted to be regulated by the test compounds (*Pa*>*Pi*), using either the down‐regulation or up‐regulation model. This analysis focused specifically on parkinsonian syndromes listed in the DisGeNET database (Barcelona, Spain). The odds ratio was used to reflect the strength of association between the predicted genes and the disease, while the *p*‐value indicated the statistical significance of this association. The adjusted *p*‐value (adj.*p*) was calculated using the Benjamini–Hochberg procedure to correct for multiple testing.

### 2.4 The in silico Predictions About in vivo LD_50_ of HBP2–3

With the in silico tool GUSAR (Department for Bioinformatics, Institute of Biomedical Chemistry of the Russian Academy of Medical Sciences, Moscow, Russia) (Lagunin et al. 2011), we conducted the predictions of LD_50_ values for acute toxicity in rats with four types of administration (oral, intravenous, intraperitoneal, subcutaneous, inhalation). Using the in silico tool DL‐AOT (Center for Quantitative Biology, Academy for Advanced Interdisciplinary Studies, Peking University, Beijing, China) (Xu et al. 2017), we forecasted LD_50_ for acute oral toxicity. The model type of DL‐AOT was “deepAOT‐R and deepAOT‐C”

### 2.5 The in vivo Zebrafish Eleutheroembryo Model

According to the methods from the previous studies (Feng et al. 2014; Huang et al. 2014), we maintained the zebrafish (the AB strain of wild‐type). Animal experiments have been conducted in accordance with the Guiding Principles in Animal Care and approved by the Kaohsiung Medical University Animal Care and Use Committee (approval no. 110138). After natural spawning, we collected the embryos, staged them by the standard criteria, and cultured them at 28.5°C in Hank's buffer. There was no further nutritional maintenance for the embryos since they could obtain nourishment from their attached yolk sacs. We initiated the treatments on the embryos with the test compounds at 9 h post fertilization (hpf) (with the solution refreshed once daily), and recorded the death events. We prepared the vehicle group (1‰ dimethyl sulfoxide [DMSO] in Hank's buffer).

### 2.6 In Silico Assessments of the Toxicological Properties of HBP2–3

The toxicological profiles of the test compounds were evaluated using a range of in silico tools. ADMETlab 3.0 (Xiangya School of Pharmaceutical Sciences, Central South University, Hunan, China) (Fu et al. 2024) was employed to predict toxicophore rules, general toxicity parameters, and potential interactions with Tox21 pathways. In addition, the TISBE platform (Department of Pharmacy‐Drug Sciences, University of Bari Aldo Moro, Bari, Italy) (Mastrolorito et al. 2024) was used to assess developmental toxicity, while embryoTox (Structural Biology and Bioinformatics, Department of Biochemistry, University of Melbourne, Victoria, Australia) (Aljarf et al. 2023) was utilized to predict teratogenic potential.

### 2.7 Analysis of the Potential Cytotoxicity of HBP2–3 in IMR‐32 Cells

Human neuroblastoma IMR‐32 cells were seeded into 96‐well plates at a density of 3 × 10^4^ cells per well and incubated for 24 h at 37°C in 5% CO_2_. At 24 h of treatment with either vehicle (1% dimethyl sulfoxide [DMSO]) or HBP2–3, cell viability was assessed. A total of 10 μL of AlamarBlue reagent (catalog no. 786–922, Invitrogen, Thermo Fisher Scientific, Massachusetts, USA) was added to each well, and fluorescence was measured using an ELISA reader at 595 nm. Cell viability was calculated using the following formula to assess the cytotoxicity of the test compounds. Formula: V = [(OD_Exp_−OD_Blank_)/(OD_vehicle_−OD_Blank_)] × 100%; V = cell viability (%); OD_Exp_ = optical density of the experimental group; OD_Blank_ = optical density of the blank group; OD_vehicle_ = optical density of the vehicle group. In this assay, the experimental group refers to either the vehicle group or the HBP2–3 group.

### 2.8 In Silico Predictions for the Potential Effects of HBP2–3 on Protein Expression

The potential effects of HBP2 and HBP3 on gene expression profiles were predicted using the in silico tool DIGEP‐Pred 2.0 (Ivanov et al. 2024), based on protein expression data from the CTD. Predictions were generated using the upregulation or downregulation models, with no restriction applied to *Pa* cutoff. This analysis specifically focused on the potential upregulation of caspase‐3 and downregulation of Bcl‐2 at the protein level by HBP2–3, as reported in the CTD. The Invariant Accuracy of Prediction (IAP) refers to the average prediction accuracy across the entire PASS training set, as evaluated by leave‐one‐out cross‐validation.

### 2.9 Western Blotting Analysis

Western blotting was performed based on a previously described method (Lee et al. 2021) to evaluate the possible effects of HBP2–3 on protein expression in the in vitro model. Cells from each group were cultured in 6‐cm dishes. After harvesting, each sample was lysed using an equal volume of lysis buffer. Protein concentrations were determined using the Bradford assay and adjusted to the same level. Equal amounts of protein (40 μg per sample) were mixed with sample buffer and subjected to SDS‐PAGE. Proteins were then transferred onto activated PVDF membranes. The membranes were blocked with 5% non‐fat dry milk in Tris‐buffered solution. The target proteins were probed using primary antibodies, followed by incubation with horseradish peroxidase‐conjugated secondary antibodies. Chemiluminescence was performed using reagents from Millipore (Merck KGaA, Darmstadt, Germany). Images were captured using the UVP BioChemi Imaging System (UVP LLC, California, USA) and analyzed using LabWorks 4.0 software (UVP). The expression of target proteins was normalized to β‐actin as the internal control. Three primary antibodies were used for overnight incubation at 4°C: active caspase‐3 with 1:500 dilution (catalog no. A11021; rabbit monoclonal antibody, ABclonal, Massachusetts, USA), Bcl‐2 with 1:1000 dilution (catalog no. A19693; rabbit monoclonal antibody, ABclonal), and β‐actin with 1:3000 dilution (catalog no. A5441; mouse monoclonal antibody, Sigma‐Aldrich, Merck KGaA, Darmstadt, Germany). We selected two secondary antibodies: goat anti‐rabbit IgG (H + L) antibody for active caspase‐3 and Bcl‐2 (with 1:10000 dilution for 1 h at 37°C; catalog no. 31460, Invitrogen), and goat anti‐mouse IgG (H + L) antibody for β‐actin (with 1:10000 dilution for 1 h at 25°C; catalog no. 31430, Invitrogen).

### 2.10 Data, Statistical Analysis, and Other Methodologies

**FIGURE 1:**
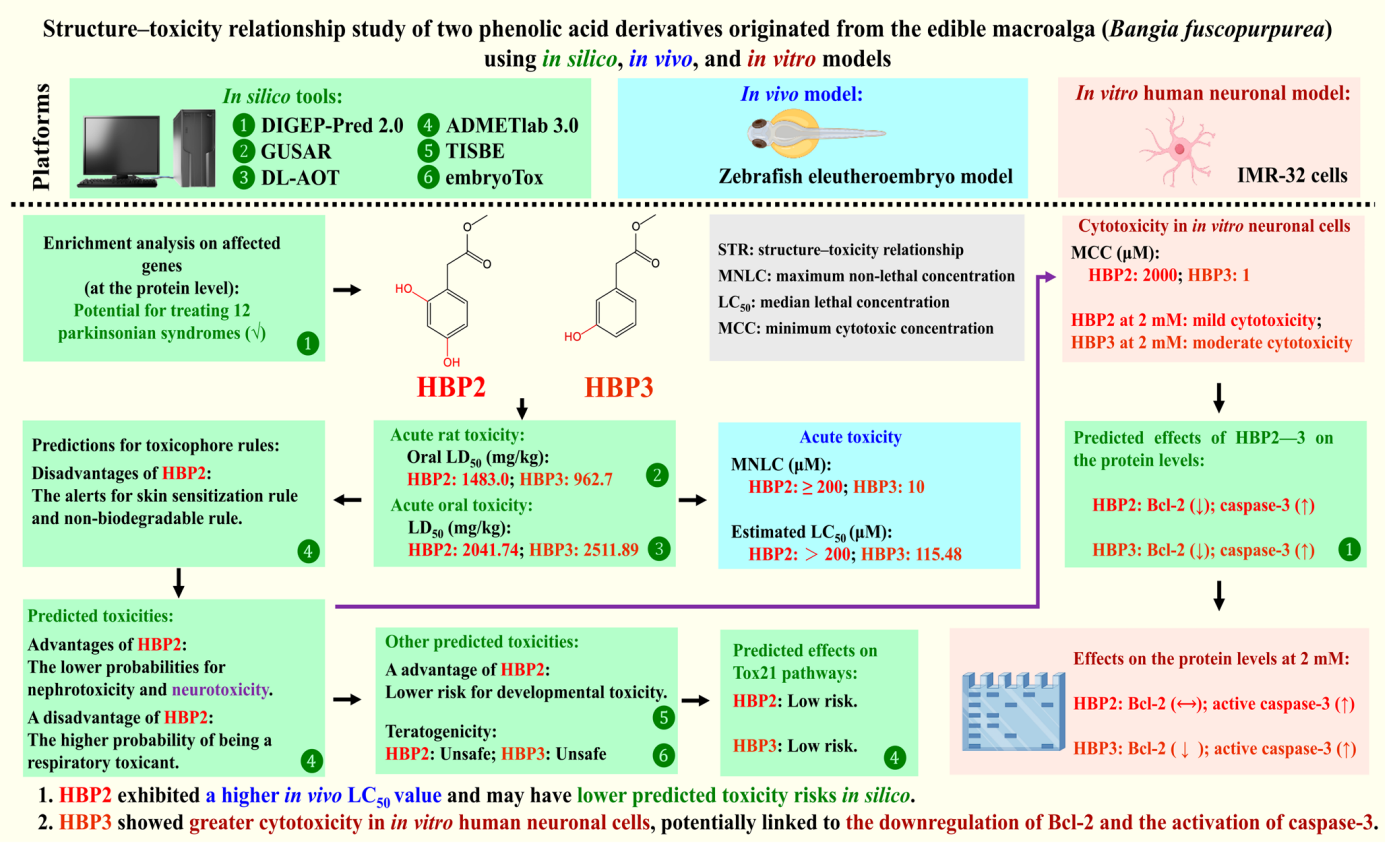
The summary figure of this study. HBP2 exhibited a higher in vivo LC_50_ value and may have lower predicted toxicity risks in silico. HBP3 showed greater cytotoxicity in in vitro human neuronal cells, potentially linked to the downregulation of Bcl‐2 and the activation of caspase‐3.

All data are presented as means ± standard error of the mean (SEM). One‐way analysis of variance (ANOVA), followed by Duncan's multiple range test, was used to assess statistical differences between groups (Dal‐Cim et al. 2012). A value of *p* < 0.05 was considered statistically significant. Based on the modified evaluation criteria for in vitro cytotoxicity (Dahl et al. 2006), the cytotoxicity of the test compound was classified as follows: cell viability > 90% was considered non‐cytotoxic, 60%–90% as mildly cytotoxic, 30%–59% as moderately cytotoxic, and < 30% as severely cytotoxic. Adopting the concept of the therapeutic index from a previous study (Greenberg et al. 2016), the therapeutic index in this study was defined as the ratio of the lowest concentration that induced toxicity to the concentration that elicited a therapeutic effect. The chemical structures of HBP2–3 were drawn using the structural editor InDraw (Integle, Shanghai, China). In addition, the illustrations in the graphical abstract and Figure 1 were created using the Figdraw platform (ID: OIWPTa9922; Figdraw 2.0; Home for Researchers, Zhejiang, China).

## 3 Results

### 3.1 Therapeutic Potential of HBP2–3 in Parkinsonian Syndromes

After conducting the literature search and to the best of our knowledge, we have confirmed that only our previous 2024 study demonstrated the neuroprotective effects of HBP2–3 in the in vitro model of Parkinson's disease (Huang et al. 2024). With the in silico tool DIGEP‐Pred 2.0, we performed the enrichment analysis on genes (at the protein level) affected by the test compound (with *Pa* > *Pi*) under the down‐regulation model (or the up‐regulation model) for parkinsonian syndromes from the DisGeNET database. In enrichment analysis, the disease that had the adj.*p* < 0.05 and was associated with at least two predicted genes for the test compound could be regarded as significantly enriched (Ivanov et al. 2024). These predictions from the two models (down‐regulation and up‐regulation models) suggest that HBP2–3 may have potential for treating 12 parkinsonian syndromes (Table S1). Both HBP2 and HBP3 were predicted to modulate genes associated with multiple forms of parkinsonian syndromes under both downregulation and upregulation models. Notably, the adjusted *p*‐values for most associations were < 0.05, indicating statistically significant enrichment.

### 3.2 Lethal Dose Estimation of HBP2–3 Using in silico Tools and the in vivo Zebrafish Eleutheroembryo Model

**FIGURE 2:**
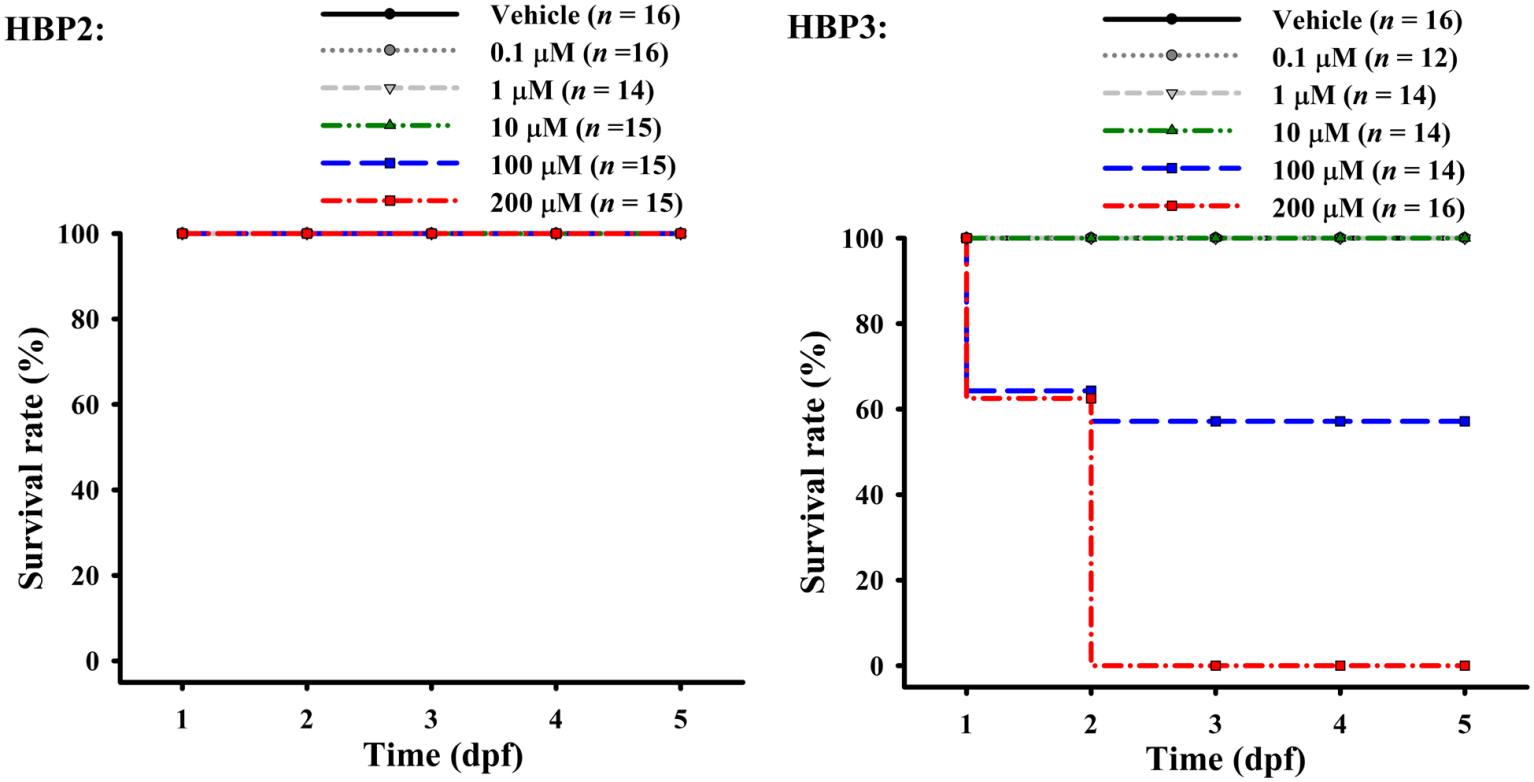
Effects of HBP2–3 on survival rate in the in vivo zebrafish eleutheroembryo model. From 1 dpf to 5 dpf, HBP2 (0.1–200 μM) did not cause death (we could not get LC_50_), and HBP3 (0.1–10 μM) did not cause death. On the other hand, the data of the lethal rates from HBP3 groups have a trendline with the *R* ^2^ value of 0.99884 and an equation of *y* = 0.0007*x* ^2^ + 0.3601*x*−0.9203, and the estimated LC_50_ of HBP3 is 115.48 μM.

We predicted four types of acute rat toxicity LD_50_ of HBP2–3 by the in silico tool GUSAR, and predicted their acute oral toxicity in rats using the in silico tool DL‐AOT (Table 1). The GUSAR tool estimated the acute toxicity LD_50_ values (mg/kg) of HBP2–3 in rats via four administration routes (intraperitoneal (IP), intravenous (IV), oral, and subcutaneous (SC)). The prediction by GUSAR showed that compared to HBP3, HBP2 had higher IV LD_50_, oral LD_50_, and SC LD_50_, suggesting lower acute toxicity of HBP2 through these administration routes. In the in vivo zebrafish eleutheroembryo model from 1 to 5 days post fertilization (dpf) (Figure 2), HBP2 (0.1–200 μM) did not induce mortality, and its median LC (LC_50_) could not be determined. No mortality was observed for HBP3 (0.1–10 μM), and its LC_50_ was 115.48 μM. Maximum non‐lethal concentration (MNLC) of HBP2 was ≥ 200 μM, and that of HBP3 was 10 μM.

**TABLE 1:** The in silico predictions of HBP2–3 for in vivo acute toxicity.

No.	Property	Comment	HBP2	HBP3
1	[A]: Acute rat toxicity	Rat IP LD_50_ (mg/kg)	511.2 (in AD)	587.8 (in AD)
			Class 5 (in AD)	Class 5 (in AD)
2		Rat IV LD_50_ (mg/kg)	206.8 (in AD)	135.9 (in AD)
			Class 4 (in AD)	Class 4 (in AD)
3		Rat oral LD_50_ (mg/kg)	1483.0 (in AD)	962.7 (in AD)
			Class 4 (in AD)	Class 4 (in AD)
4		Rat SC LD_50_ (mg/kg)	1024.0 (in AD)	746.8 (in AD)
			Class 5 (in AD)	Class 4 (in AD)
5	[B]: Acute oral toxicity	LD_50_ (mg/kg) (log(LD_50_))	2041.74 (3.31)	2511.89 (3.40)
		Toxicity class (probability) Toxicity class: (1) danger/poison; (2) warning; (3) caution; (4) none required	Class 3 (1.00)	Class 3 (1.00)

*Note:* [A]: GUSAR; acute rodent toxicity classification of chemicals by OECD project. IP: intraperitoneal route of administration; IV: intravenous route of administration; Oral: oral route of administration; SC: subcutaneous route of administration. In AD: compound falls in applicability domain of models; Out of AD: compound is out of applicability domain of models. [B]: DL‐AOT.

### 3.3 The Predictions for the Toxicological Properties of HBP2–3

We used the in silico tool ADMETlab 3.0 to predict the analysis for the toxicophore rules (Table S2), toxicities (Table 2), and possible effects on the Tox21 pathways (Table S3). The resulting predictions were annotated with four color labels indicating performance levels: green (excellent), yellow (medium), red (poor), and gray (alert). Except for the skin sensitization rule and biodegradability, HBP2 shared the same in silico predictions as HBP3 (Table S2). The overall predicted outcomes of HBP2 were largely consistent with those of HBP3, except for three notable differences in respiratory toxicity, drug‐induced nephrotoxicity, and drug‐induced neurotoxicity (Table 2). As shown in Table S3, both HBP2 and HBP3 exhibited very low predicted probabilities of activity (all < 0.2) across all 12 Tox21 pathways, suggesting a minimal likelihood of pathway‐specific endocrine disruption or cellular stress induction. These predictions support the low systemic toxicity profiles of HBP2 and HBP3 and offer mechanistic insights into their potential safety. In addition, we used two in silico tools (TISBE and embryoTox) to predict their developmental toxicity and teratogenicity, respectively. HBP3 exhibited a higher predicted risk of developmental toxicity than HBP2, and both compounds were predicted to be unsafe for teratogenicity (Table 2). Compared with HBP3, HBP2 had the advantages: (a) the lower probabilities for nephrotoxicity and neurotoxicity, and (b) the lower risk for developmental toxicity. HBP2 had the weaknesses: (a) the alerts for the skin sensitization rule and non‐biodegradable rule, (b) the higher probability of being a respiratory toxicant.

**TABLE 2:** In Silico predictions for the general and environmental toxicities of HBP2–3.

No.	Property	Comment	HBP2	HBP3
I. Toxicities				
1	hERG blockers	[A]: The probability of being blockers IC_50_ > 10 μM or < 50% inhibition at 10 μM: 0; IC_50_ ≤ 10 μM or ≥ 50% inhibition at 10 μM: 1	0.043	0.071
2	hERG blockers (10 μM)	[A]: The probability of being blockers IC_50_ > 10 μM: 0; IC_50_ ≤ 10 μM: 1	0.602	0.551
3	DILI	[A]: Drug induced liver injury (DILI). The probability of being toxic. No risk: 0; high risk: 1	0.103	0.234
4	Ames toxicity	[A]: The probability of being toxic. Negative: 0; positive: 1	0.458	0.46
5	Rat oral acute toxicity	[A]: The probability of being toxic. Low toxicity (> 500 mg/Kg): 0; high toxicity (< 500 mg/Kg): 1	0.497	0.472
6	FDAMDD	[A]: FDA maximum (recommended) daily dose (FDAMDD). The probability of being positive. Negative: 0; positive: 1	0.209	0.188
7	Skin sensitization	[A]: The probability of being toxic. Non‐sensitizer: 0; sensitizer: 1	0.845	0.861
8	Carcinogenicity	[A]: The probability of being toxic. Non‐carcinogens: 0; carcinogens: 1	0.386	0.423
9	Eye corrosion	[A]: The probability of being corrosives. Negative: 0; positive: 1	0.777	0.721
10	Eye irritation	[A]: The probability of being irritants. Negative: 0; positive: 1	0.994	0.987
11	Respiratory	[A]: The probability of being respiratory toxicants. Negative: 0; positive: 1	0.847	0.564
12	Human hepatotoxicity	[A]: The probability of being toxic. Negative: 0; positive: 1	0.325	0.305
13	Drug‐induced nephrotoxicity	[A]: The probability of being toxic. Negative: 0; positive: 1	0.123	0.306
14	Drug‐induced neurotoxicity	[A]: The probability of being toxic. Negative: 0; positive: 1	0.266	0.518
15	Ototoxicity	[A]: The probability of being toxic. Negative: 0; positive: 1	0.113	0.141
16	Hematotoxicity	[A]: The probability of being toxic. Negative: 0; positive: 1	0.101	0.203
17	Genotoxicity	[A]: The probability of being toxic. Negative: 0; positive: 1	0.541	0.311
18	RPMI‐8226 immunitoxicity	[A]: The probability of being toxic. Negative: 0; positive: 1	0.03	0.028
19	A549 cytotoxicity	[A]: The probability of being toxic. Negative: 0; positive: 1	0.032	0.024
20	Hek293 cytotoxicity	[A]: The probability of being toxic. Negative: 0; positive: 1	0.26	0.121
21	Developmental toxicity	[B]: The score higher than 0.5: toxic. The prediction of HBP2 is considered not reliable, but that of HBP3 is considered reliable Already in dataset of TISBE: false for HBP2 and HBP3	Toxicant (0.82)	Toxicant (0.84)
22	Teratogenicity	[C]: Safety profile (confidence level) Confidence: HBP2 (0.838); HBP3 (0.838)	Unsafe (High)	Unsafe (High)
II. Environmental toxicities				
1	BCF	[A]: Bioconcentration factors are used for considering secondary poisoning potential and assessing risks to human health via the food chain. The unit is −log_10_ [(mg/L)/(1000 × MW)]	0.743	0.598
2	IGC_50_	[A]: *Tetrahymena pyriformis* 50% growth inhibition concentration. The unit is −log_10_ [(mg/L)/(1000 × MW)]	2.743	2.418
3	LC_50_DM	[A]: 48‐h *daphnia magna* 50% lethal concentration The unit is −log_10_ [(mg/L)/(1000 × MW)]	3.717	3.529
4	LC_50_FM	[A]: 96‐h fathead minnow 50% lethal concentration The unit is −log_10_ [(mg/L)/(1000 × MW)]	3.073	2.838

*Note:* [A]: ADMETlab 3.0; [B]: TISBE; [C]: embryoTox.

### 3.4 Comparison of the in vitro Cytotoxicity of HBP2–3 in Human Neuronal Cells

**FIGURE 3:**
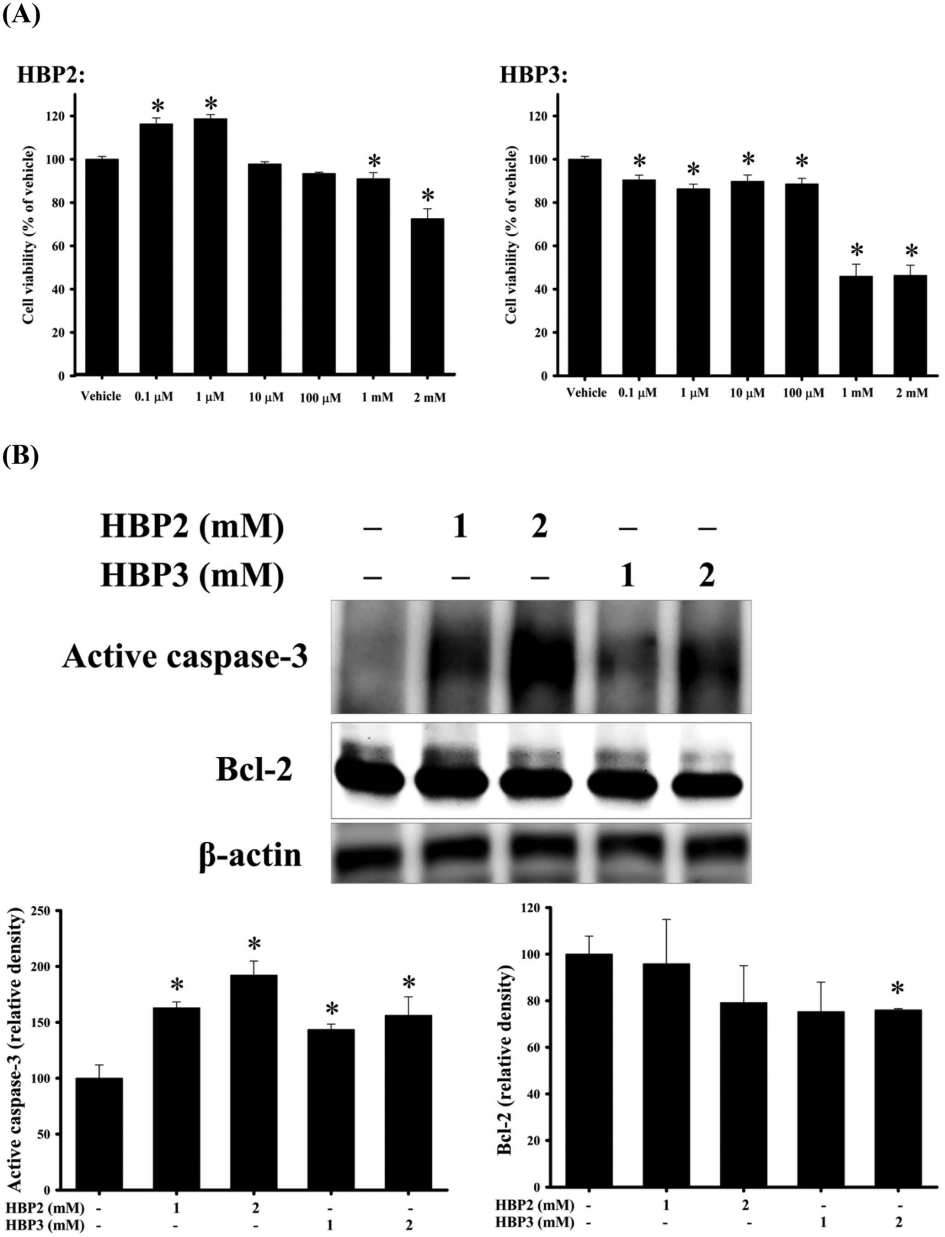
In vitro effects of HBP2–3 on the human neuronal IMR‐32 model after 24 h of treatment. (A) Effects of HBP2–3 on cell viability (*n* = 4). (B) Effects of HBP2–3 on protein expression levels (*n* = 3). The lowest cytotoxic concentrations of the compounds: 2 mM for HBP2, and 1 μM for HBP3. At 2 mM, HBP3 induced the upregulation of active caspase‐3 and the downregulation of Bcl‐2, but HBP2 only caused the upregulation of active caspase‐3. *p < 0.05 compared with the vehicle group.

The results of cell viability analysis in the human neuronal IMR‐32 model after 24 h of treatment (Figure 3A) revealed that the minimum cytotoxic concentration of each compound (defined as < 90% cell viability, indicating mild cytotoxicity) was 2 mM for HBP2 and 1 μM for HBP3. HBP2 at 2 mM exhibited mild cytotoxicity, whereas HBP3 at 1–2 mM induced moderate cytotoxicity. Caspase‐3 can be regarded as an apoptotic marker (Abas et al. 2025; Ma et al. 2025; Malik et al. 2025; Peng et al. 2025). Bcl‐2 serves as an anti‐apoptotic protein (Xu et al. 2021). We used the in silico tool DIGEP‐Pred 2.0 to predict the potential effects of HBP2–3 on the protein expression of caspase‐3 and Bcl‐2 (Table S4). Only predictions with *Pa* greater than *Pi* were considered relevant (possible events) (Ivanov et al. 2024). A *Pa* threshold of 0.500 was applied to both the upregulation and downregulation models. HBP2–3 may upregulate caspase‐3 and downregulate Bcl‐2 at the protein level. Western blotting analysis of in vitro IMR‐32 cells treated with 2 mM of the compounds revealed that HBP3 induced the upregulation of active caspase‐3 and downregulation of Bcl‐2, whereas HBP2 caused only the active caspase‐3 upregulation (Figure 3B).

## 4 Discussion

### 4.1 The Possible Bioactivities of HBP2–3 Originated From *Bangia fuscopurpurea*: The in silico Studies for Their Possible Potential for Treating Parkinsonian Syndromes

A previous 2023 study successfully identified the structures of HBP2–3 in the ethanol extracts of *Bangia fuscopurpurea* (Chang et al. 2023). During the early phase of drug discovery, searching for the active compounds for a target could still be both a high time‐ and resource‐intensive project, and the in silico prediction of bioactivity could refine the process for compound screening in a lesser and more subtle way (Fredin Haslum et al. 2024). The number of possible literatures for HBP2–3 in PubChem was less than 7 (Table 3). The above number of possible literatures partly reflects the relative lack of research on HBP2–3, especially for their possible biological activities and toxicological properties. The predictions at the protein level from the tool DIGEP‐Pred 2.0 suggested that HBP2–3 may have potential for treating parkinsonian syndromes (Table S1). The above predictions about HBP2–3 may be consistent with our previous 2024 study that indicated their in vitro neuroprotective effects for the model of Parkinson's disease (Huang et al. 2024). However, the appropriate starting experimental concentration range for HBP2–3 in animal models remained unclear, and it was also uncertain which compound may present the lower toxicity risk.

**TABLE 3:** The summary of the previous research and the experimental results in the present study of HBP2–3.

Items	Compounds		HBP2	HBP3
Previous studies	Structures		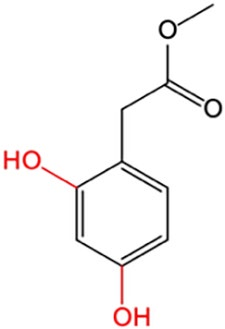	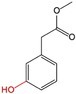
	Current status		Originated from the edible macroalga *Bangia fuscopurpurea*	
	The numbers of possible related literatures	WOS	3	7
		PubMed; PubChem	10; 1	2; 6
	Bioactivities		in vitro neuroprotection (therapeutic index: 10,000; MPC: 100 nM) (Huang et al. 2024). Free radical scavenging property (Nahar et al. 2005)	in vitro neuroprotection (therapeutic index: 10,000; MPC: 10 nM) (Huang et al. 2024)
The present study	(1) The potential for treating 12 parkinsonian syndromes	in silico model (DIGEP‐Pred 2.0)	Yes	Yes
	(2) Acute toxicity: Rat oral LD_50_ (mg/kg)	in silico model (GUSAR)	1483.0 mg/kg	962.7 mg/kg
	(3) Acute toxicity: Maximum non‐lethal concentration (MNLC)	in vivo zebrafish eleutheroembryo model	≥ 200 μM	10 μM
	(4) Acute toxicity: LC_50_		We could not get LC_50_	Estimated LC_50_: 115.48 μM
	(5) Modified therapeutic index	in vivo MNLC in vitro MPC (Huang et al. 2024)	≥ 2000	1000
	(6) Predictions for the toxicophore rules	in silico model (ADMETlab 3.0)	Disadvantages of HBP2: The alerts for skin sensitization rule and non‐biodegradable rule	
	(7) Predicted toxicities	in silico model (ADMETlab 3.0)	Advantages of HBP2: The lower probabilities for drug‐induced nephrotoxicity and drug‐induced neurotoxicity	
			A disadvantage of HBP2: The higher probability of being a respiratory toxicant	
		in silico model (TISBE)	A advantage of HBP2: The lower risk for developmental toxicity	

	(8) Predicted effects on the Tox21 pathways	in silico model (ADMETlab 3.0)	Features of HBP2 and HBP3: Low risk	
	(9) Predicted effects on protein expression levels	in silico model (DIGEP‐Pred 2.0)	Bcl‐2 (↓); caspase‐3 (↑)	Bcl‐2 (↓); caspase‐3 (↑)
	(10) Minimum cytotoxic concentration	in vitro *human neuronal* model (IMR‐32)	2 mM	1 μM
(11) Effect of 2 mM treatment on protein expression levels	Bcl‐2 (↔); activation of caspase‐3 (↑)		Bcl‐2 (↓); activation of caspase‐3 (↑)

*Note:* Light gray: in silico model; dark gray: in vivo model. MPC: minimum protective concentration.

### 4.2 Determination of the Lethal Dose of HBP2–3 Using the in silico Model and the in vivo Zebrafish Eleutheroembryo Model

The LD_50_ test was introduced in 1927 by J. W. Trevan to estimate the dose of a test substance that produces 50% death in animals (Erhirhie et al. 2018). The LD_50_ test is usually the first test conducted for every chemical before further toxicity tests are carried out (Chinedu et al. 2013; Erhirhie et al. 2018). The LD_50_ test in animals is used for estimating the potential hazards of chemicals on humans (Erhirhie et al. 2018). For the acute toxicity, we reported that the predicted oral LD_50_ values of HBP2 (1483.0 mg/kg) and HBP3 (962.7 mg/kg) in rats by the in silico tool GUSAR (Table 1), which were consistent with the predicted oral LD_50_ values of HBP2 (1550 mg/kg) and HBP3 (1000 mg/kg) in rodents by the in silico tool ProTox‐II (Huang et al. 2024). Our in vivo zebrafish experimental results (Figure 2) align with the aforementioned in silico findings, indicating that HBP2 has lower toxicity than HBP3. In the in vivo zebrafish eleutheroembryo model, MNLC of HBP2 was ≥ 200 μM, and that of HBP3 was 10 μM. These MNLC values of HBP2–3 could be the appropriate starting experimental concentration range in the in vivo zebrafish eleutheroembryo model for the explorations of other possible bioactivities. We attempted to fit the mortality data of HBP3 in the in vivo zebrafish eleutheroembryo model (Figure 2) using a sigmoid (logistic) model; however, due to the limited number of mortality data points (only two concentrations caused lethality), the model failed to converge despite parameter constraints. This represents a limitation of the present study and highlights the need for further in vivo experiments in the future. The in vivo data of the lethal rates from HBP3 groups (0.1–10 μM) in the zebrafish eleutheroembryo model have a trendline with an equation of *y* = 0.0007x^2^ + 0.3601x−0.9203 (the *R* ^2^ value of 0.99884), and LC_50_ of HBP3 is 115.48 μM. Another limitation of this study is that we could not determine the LC_50_ for HBP2 in the zebrafish eleutheroembryo model.

### 4.3 Discrepancy Between in vivo Toxicity of HBP3 in the Zebrafish Eleutheroembryo Model and Predicted Oral LD_50_ Values From in silico Tools

The observed discrepancy between the in vivo toxicity of HBP3 in the zebrafish eleutheroembryo model and the higher oral LD_50_ values predicted by the in silico tools may be attributed to the following three factors. (1) Species and developmental stage differences: in silico tools typically estimate toxicity based on adult rodent data, whereas zebrafish eleutheroembryos are more vulnerable to chemical insults due to their immature detoxification systems and developing organs. (2) Exposure route: the predicted LD_50_ values reflect oral administration in mammals, while zebrafish embryos are exposed via aqueous immersion, potentially leading to higher local concentrations and faster uptake. (3) Model limitations: although platforms such as GUSAR and ProTox‐II offer valuable estimates, their algorithms are based predominantly on mammalian datasets and fail to account for species‐ or developmental‐stage‐specific toxicity mechanisms that are relevant in zebrafish embryos.

### 4.4 In vitro Cytotoxicity Comparison of HBP2–3 in Human Neuronal Cells

Central nervous system (CNS) toxicity was reported to account for nearly 25% of failures in drug development (Walker et al. 2018). Given the lower predicted probability of neurotoxicity for HBP2 compared to HBP3 (Table 2), an in vitro human neuronal IMR‐32 model was subsequently employed to further assess their cytotoxicity. Similar to SH‐SY5Y cells, IMR‐32 cells have been widely utilized as an in vitro neuronal model (Leung et al. 2024). In IMR‐32 cells following 24 h of treatment (Figure 3A), the minimum cytotoxic concentration was 2 mM for HBP2 and 1 μM for HBP3. Caspase‐3 activation represents both a crucial event and a central mediator in neuronal programmed cell death (D'Amelio et al. 2012). In addition to the activation of caspase‐3, neuronal apoptosis was reported to be associated with the downregulation of Bcl‐2 (Liu et al. 2013). In silico model showed that HBP2–3 may upregulate caspase‐3 and downregulate Bcl‐2 at the protein level (Table S4). In in vitro human neuronal cells at 2 mM, HBP3 exhibited higher cytotoxicity accompanied by increased caspase‐3 activation and decreased Bcl‐2 protein expression, whereas HBP2 showed lower cytotoxicity without affecting Bcl‐2 expression (Figure 3B). In agreement with the findings from zebrafish eleutheroembryo experiments, HBP2 showed lower cytotoxicity in human neuronal cells compared to HBP3.

### 4.5 Comparison of HBP2 and HBP3 Using the Modified Therapeutic Index

In the in vitro model of Parkinson's disease, HBP2 and HBP3 had the same value (10,000) for the therapeutic index (Huang et al. 2024). With the definition of the minimum inhibitory concentration (MIC; the lowest concentration of the test compound for the specific bioactivity) (Van Dijck et al. 2018), our previous study found that the minimum protective concentration (MPE) for the in vitro model of Parkinson's disease was 0.1 μM for HBP2 and 10 nM for HBP3 (Huang et al. 2024). Based on the modified therapeutic index calculation, we found that the value was ≥ 2000 for HBP2 and 1000 for HBP3 (Table 3). In the in silico models, HBP2 generally might have lower toxicity risks than HBP3 (Table 3). HBP2, derived from the seeds of *Ilex aquifolium* , has been reported to possess free radical scavenging activity (Nahar et al. 2005). In addition, HBP2 at 0.1–1 μM significantly increased cell viability (Figure 3A). Our present study suggested that HBP2 had a higher development potential for future studies in the field of neuroprotection than HBP3. There remains a risk that in silico tools may not fully predict the toxicological properties of HBP2–3.

### 4.6 The STR Studies of HBP2–3

By reviewing the previous studies about the clinical trials (from 2010 to 2017), Sun et al. concluded that the toxicity (unmanageable or unexpected) accounted for 30% of the possible reasons for clinical drug development failures (Sun et al. 2022). For developing natural products into medicines, one of the key functions of structural modifications could be the decrease of toxic effects (Guo 2017). The STR analysis of the target compounds could provide a theoretical foundation for future research on their structural modifications for lower toxicity (Guha 2013). Based on the in vivo, in vitro, and in silico data (Table 3), this study suggested that HBP2 exhibited a higher LC_50_ in the in vivo model, lower cytotoxicity in in vitro neuronal cells, and potentially lower overall toxicity risks (Figure 1). These data from in vivo and in silico models may be associated with the increase of the degree of hydroxylation. We suggested that this feature could be a potential topic for future STR studies related to this class of phenolic acid derivatives. In contrast, when we compared the predicted protein‐level downregulation profiles of HBP2 (1148 gene entries) and HBP3 (1149 gene entries) using the in silico tool DIGEP‐Pred 2.0 (Table S1), we found that the two compounds affected the same set of 1148 proteins. Notably, only one protein, synaptophysin, was uniquely affected by HBP3 (Pa = 0.118 > Pi = 0.044; IAP = 0.993). Synaptophysin has been found in neurons at synaptic sites, and it could be used as a marker of synapses (Utz et al. 2021). Two review articles have reported that substance‐induced neurotoxicity is associated with the downregulation of synaptophysin (Aschner et al. 2024; Santos et al. 2020). We suggest that future studies further elucidate the potential differential effects of HBP2–3 on synaptophysin at the protein level using neuronal cell models.

### 4.7 Limitations of the Application of the DIGEP‐Pred 2.0 Platform in This Study

DIGEP‐Pred 2.0 is a ligand‐based virtual screening tool that predicts compound‐induced gene and protein expression changes based on structure–activity relationship (SAR) models (Ivanov et al. 2024). These SAR models were trained using experimentally measured drug‐induced expression data from the Connectivity Map (CMap) and the Comparative Toxicogenomics Database (CTD). Specifically, the CMap dataset includes transcriptomic responses from non‐neuronal human cancer cell lines, namely MCF7 (breast adenocarcinoma), PC3 (prostate carcinoma), and HL60 (promyelocytic leukemia). We fully acknowledge that the cell lines used in the SAR training data are not of neuronal origin. However, our application of DIGEP‐Pred 2.0 was not intended to model neuron‐specific expression directly, but rather to identify potential compound–gene interactions and enriched pathways that may be mechanistically associated with neurotoxicity and Parkinsonian syndromes, which are the focus of our study. These predictions served as an exploratory reference to support biological interpretation. Additionally, we did not rely on these in silico results in isolation. Instead, we tried to partially complement them with experimental validation using the in vitro human neuronal IMR‐32 model (Figure 3B), aiming to cross‐verify two toxicity‐related predictions for HBP2–3, namely the upregulation of caspase‐3 and the downregulation of Bcl‐2 at the protein level (Table S4).

### 4.8 Future Studies on HBP2

We reported that HBP2–3 exerted neuroprotective effects in the in vitro neuronal SH‐SY5Y model of Parkinson's disease (Huang et al. 2024). These in silico findings from Table S1 suggest a potential mechanistic link between HBP2 and HBP3 and pathways involved in Parkinson's disease, warranting further i*n vivo* experimental validation in the future. The in vivo zebrafish eleutheroembryo model of Parkinson's disease induced by 6‐hydroxydopamine (6‐OHDA) (Feng et al. 2014) has been cited over 100 times. This study primarily referred to the dosing schedule and administration method of the aforementioned zebrafish PD model, and we initiated the treatments on the embryos with the test compounds at 9 hpf (with the solution refreshed once daily to 5 dpf). One of the primary aims of this study was to determine the safe concentration range of the compound based on zebrafish mortality data. These findings will serve as a reference for selecting appropriate experimental concentrations in future studies employing zebrafish behavioral assays as safety indicators to further evaluate the compound's potential effects on Parkinson's disease. According to OECD Test Guideline 236 (OECD 2025), which is currently considered the gold standard for zebrafish embryo toxicity assays, embryos should be exposed to test compounds no later than 3 hpf, with daily renewal of the test solution until 96 hpf. One of the limitations of the zebrafish eleutheroembryo model used in this study is the lack of adherence to standardized OECD testing guidelines, which may affect the regulatory applicability of the findings. Taken together, our findings indicate that HBP2 holds greater potential for further development. We therefore recommend that future studies consider 200 μM as the initial experimental concentration in zebrafish eleutheroembryo models.

## 5 Conclusions

Compared to HBP3, our present study demonstrates that HBP2 exhibits a higher LC_50_ in the in vivo zebrafish eleutheroembryo model, lower cytotoxicity in in vitro human neuronal cells, and potentially lower toxicity risks predicted by in silico tools. These toxicological characteristics may be linked to a higher degree of hydroxylation in HBP2. This study contributes to a better understanding of HBP2 and HBP3, originating from *Bangia fuscopurpurea*, from the perspective of molecular nutrition and STR analysis.

## Author Contributions

**Shi‐Ying Huang:** conceptualization (equal), formal analysis (equal), investigation (equal), methodology (equal), writing – original draft (equal), writing – review and editing (equal). **Guiling Li:** conceptualization (equal), formal analysis (equal), investigation (equal), methodology (equal), writing – original draft (equal), writing – review and editing (equal). **Yi‐Jia Shih:** formal analysis (supporting), investigation (supporting), methodology (supporting), writing – original draft (supporting). **Chang‐Wei Hsieh:** formal analysis (supporting), investigation (supporting), writing – original draft (supporting). **Yun‐Sheng Lin:** formal analysis (supporting), investigation (supporting), writing – original draft (supporting). **Jingwen Liu:** investigation (supporting), writing – original draft (supporting). **Tao Sun:** conceptualization (equal), formal analysis (equal), investigation (equal), methodology (equal), writing – original draft (equal), writing – review and editing (equal). **Chien‐Wei Feng:** conceptualization (equal), formal analysis (equal), investigation (equal), methodology (equal), writing – original draft (equal), writing – review and editing (equal).

## Conflicts of Interest

The authors declare no conflicts of interest.

## Supporting information


**Table S1:** In Silico enrichment analysis of the predicted effects of HBP2–3 on proteins associated with parkinsonian syndromes in the DisGeNET database.
**Table S2:** In Silico predictions of HBP2–3 based on toxicophore rules.
**Table S3:** In Silico predictions for possible effects of HBP2–3 on Tox21 pathways.
**Table S4:** In Silico predicted effects of HBP2–3 on the protein levels of caspase‐3 and Bcl‐2.

## Data Availability

The data are available from the authors upon reasonable request.
