# Supplementary material for: Determination of the Lethal Concentrations of Two Phenolic Acid Derivatives Originated From the Edible Red Marine Macroalga (Bangia fuscopurpurea) Using the In Vivo Zebrafish Eleutheroembryo Model and Their In Silico Structure–Toxicity Relationship Study
Source: Food Sci Nutr. 2026 Jan 21;14(1):e71182. doi: 10.1002/fsn3.71182 (PMC12824449; doi:10.1002/fsn3.71182)
Supplement: Supplementary file 1 — Table S1: In Silico enrichment analysis of the predicted effects of HBP2–3 on proteins associated with parkinsonian syndromes in the DisGeNET database. Table S2: In Silico predictions of HBP2–3 based on toxicophore rules. Table S3: In Silico predictions for possible effects of HBP2–3 on Tox21 pathways. Table S4: In Silico predicted effects of HBP2–3 on the protein levels of caspase‐3 and Bcl‐2. [file FSN3-14-e71182-s001.docx]

**Supplementary Results**

**Determination of the lethal concentrations of two phenolic acid derivatives originated from the edible red marine macroalga (*Bangia fuscopurpurea*) using the *in vivo* zebrafish eleutheroembryo model and their *in silico* structure–toxicity relationship study**

**Running Head: *In vivo* LC of HBP2–3 and *in silico* STR study**

**Shi-Ying Huang^1,#^, Guiling Li^1,2,#^, Yi-Jia Shih^3^, Chang-Wei Hsieh^4,5,6^, Yun-Sheng Lin^7^, Jingwen Liu^1^, Tao Sun^8,9,*^, Chien-Wei Feng^10,11,*^**

**^1^** College of Ocean Food and Biological Engineering, Jimei University, Xiamen 361021, China.

**^2^** Fujian Marine Functional Food Engineering Technology Research Center, Xiamen 361021, China.

**^3^** Sustainable Ocean Governance Center, National Sun Yat-sen University, Kaohsiung 804201, Taiwan.

**^4^** Department of Food Science and Biotechnology, National Chung Hsing University, Taichung 402202, Taiwan.

**^5^** Department of Food Science, National Ilan University, Yilan 260007, Taiwan.

**^6^** Department of Medical Research, China Medical University Hospital, Taichung 404333, Taiwan.

**^7^** Department of Food Science, National Pingtung University of Science and Technology, Pingtung 912301, Taiwan.

**^8^** Center for Precision Medicine, Huaqiao University, Xiamen 361021, China.

**^9^** School of Medicine and School of Biomedical Sciences, Huaqiao University, Xiamen 361021, China.

**^10^** Department of Obstetrics and Gynecology, Kaohsiung Medical University Hospital, Kaohsiung Medical University, Kaohsiung, 807378, Taiwan.

**^11^** Center for Cancer Research, Kaohsiung Medical University, Kaohsiung, 807378, Taiwan.

**#These authors contributed equally.**

*** Corresponding Authors.**

**Supplementary Table S1. *In silico* enrichment analysis of the predicted effects of HBP2–3 on proteins associated with parkinsonian syndromes in the DisGeNET database.**

| **I. Downregulation model (*Pa* > *Pi*)** | | | | | | | | | | | | | |
| --- | --- | --- | --- | --- | --- | --- | --- | --- | --- | --- | --- | --- | --- |
| No. | Name  (disease ID) | **HBP2** (1148 entries of genes) | | | | | | **HBP3** (1149 entries of genes) | | | | | |
|  |  | n | N | Odds ratio | p-value | adj.p | *adj.p* ＜0.05 | n | N | Odds ratio | p-value | adj.p | *adj.p*＜0.05 |
| 1 | C0030567 | 46 | 127 | 7.628 | 2.22e-22 | 3.19e-21 | Yes | 46 | 127 | 7.621 | 2.30e-22 | 3.27e-21 | Yes |
| 2 | C0030569 | 3 | 12 | 5.091 | 3.07e-2 | 3.69e-2 | Yes | 3 | 12 | 5.087 | 3.07e-2 | 3.70e-2 | Yes |
| 3 | C0242422 | 31 | 85 | 7.591 | 1.35e-15 | 1.12e-14 | Yes | 31 | 85 | 7.585 | 1.38e-15 | 1.14e-14 | Yes |
| 4 | C0751414 | 3 | 12 | 5.091 | 3.07e-2 | 3.69e-2 | Yes | 3 | 12 | 5.087 | 3.07e-2 | 3.70e-2 | Yes |
| 5 | C0751415 | 3 | 12 | 5.091 | 3.07e-2 | 3.69e-2 | Yes | 3 | 12 | 5.087 | 3.07e-2 | 3.70e-2 | Yes |
| 6 | C0752097 | 9 | 28 | 6.575 | 4.02e-5 | 8.10e-5 | Yes | 9 | 28 | 6.569 | 4.05e-5 | 8.10e-5 | Yes |
| 7 | C0752098 | 9 | 28 | 6.575 | 4.02e-5 | 8.10e-5 | Yes | 9 | 28 | 6.569 | 4.05e-5 | 8.10e-5 | Yes |
| 8 | C0752100 | 9 | 28 | 6.575 | 4.02e-5 | 8.10e-5 | Yes | 9 | 28 | 6.569 | 4.05e-5 | 8.10e-5 | Yes |
| 9 | C0752101 | 9 | 28 | 6.575 | 4.02e-5 | 8.10e-5 | Yes | 9 | 28 | 6.569 | 4.05e-5 | 8.10e-5 | Yes |
| 10 | C0752104 | 9 | 28 | 6.575 | 4.02e-5 | 8.10e-5 | Yes | 9 | 28 | 6.569 | 4.05e-5 | 8.10e-5 | Yes |
| 11 | C0752105 | 9 | 30 | 6.137 | 6.29e-5 | 1.21e-4 | Yes | 9 | 30 | 6.132 | 6.33e-5 | 1.22e-4 | Yes |
| 12 | C1868675 | 9 | 28 | 6.575 | 4.02e-5 | 8.10e-5 | Yes | 9 | 28 | 6.569 | 4.05e-5 | 8.10e-5 | Yes |
| **II. Upregulation model (*Pa* > *Pi*)** | | | | | | | | | | | | | |
| No. | Name  (disease ID) | **HBP2** (2154 entries of genes) | | | | | | **HBP3** (2155 entries of genes) | | | | | |
|  |  | n | N | Odds ratio | p-value | adj.p | *adj.p* ＜0.05 | n | N | Odds ratio | p-value | adj.p | *adj.p* ＜0.05 |
| 1 | C0030567 | 52 | 127 | 4.520 | 8.90e-16 | 9.95e-15 | Yes | 52 | 127 | 4.518 | 9.06e-16 | 1.01e-14 | Yes |
| 2 | C0030569 | 5 | 12 | 4.522 | 1.12e-2 | 1.62e-2 | Yes | 5 | 12 | 4.520 | 1.12e-2 | 1.62e-2 | Yes |
| 3 | C0242422 | 38 | 85 | 4.912 | 7.81e-13 | 6.41e-12 | Yes | 38 | 85 | 4.909 | 7.91e-13 | 6.50e-12 | Yes |
| 4 | C0751414 | 5 | 12 | 4.522 | 1.12e-2 | 1.62e-2 | Yes | 5 | 12 | 4.520 | 1.12e-2 | 1.62e-2 | Yes |
| 5 | C0751415 | 5 | 12 | 4.522 | 1.12e-2 | 1.62e-2 | Yes | 5 | 12 | 4.520 | 1.12e-2 | 1.62e-2 | Yes |
| 6 | C0752097 | 15 | 28 | 5.836 | 1.18e-6 | 4.14e-6 | Yes | 15 | 28 | 5.834 | 1.19e-6 | 4.15e-6 | Yes |
| 7 | C0752098 | 15 | 28 | 5.836 | 1.18e-6 | 4.14e-6 | Yes | 15 | 28 | 5.834 | 1.19e-6 | 4.15e-6 | Yes |
| 8 | C0752100 | 15 | 28 | 5.836 | 1.18e-6 | 4.14e-6 | Yes | 15 | 28 | 5.834 | 1.19e-6 | 4.15e-6 | Yes |
| 9 | C0752101 | 15 | 28 | 5.836 | 1.18e-6 | 4.14e-6 | Yes | 15 | 28 | 5.834 | 1.19e-6 | 4.15e-6 | Yes |
| 10 | C0752104 | 15 | 28 | 5.836 | 1.18e-6 | 4.14e-6 | Yes | 15 | 28 | 5.834 | 1.19e-6 | 4.15e-6 | Yes |
| 11 | C0752105 | 15 | 30 | 5.447 | 2.28e-6 | 7.56e-6 | Yes | 15 | 30 | 5.444 | 2.30e-6 | 7.58e-6 | Yes |
| 12 | C1868675 | 15 | 28 | 5.836 | 1.18e-6 | 4.14e-6 | Yes | 15 | 28 | 5.834 | 1.19e-6 | 4.15e-6 | Yes |

**n:** the number of down-regulated (or up-regulated) genes predicted for the test compound with *Pa* ＞ *Pi* that are associated with the particular disease (such as Parkinson disease).

**N:** the number of background genes annotated in the DisGeNET database that are associated with the particular disease.

***Pa***: probability of being active; ***Pi***: probability of being inactive; **adj.p**: Adjusted p-value.

**Name (disease ID):** **(1)** Parkinson disease (C0030567); **(2)** Secondary Parkinson disease (C0030569); **(3)** Parkinsonian disorders (C0242422); **(4)** Parkinson disease, secondary vascular (C0751414); **(5)** Atherosclerotic Parkinsonism (C0751415); **(6)** Autosomal dominant juvenile Parkinson disease (C0752097); **(7)** Autosomal dominant Parkinsonism (C0752098); **(8)** Autosomal recessive Parkinsonism (C0752100); **(9)** Parkinsonism, experimental (C0752101); **(10)** Familial Juvenile Parkinsonism (C0752104); **(11)** Parkinsonism, Juvenile (C0752105); **(12)** Parkinson disease 2, autosomal recessive juvenile (C1868675).

**Supplementary Table S2. *In silico* predictions of HBP2–3 based on toxicophore rules.**

| **No.** | **Property** | **Comment** | **HBP2** | **HBP3** |
| --- | --- | --- | --- | --- |
| 1 | Acute aquatic toxicity rule | 99 substructures: toxicity to liquid (water). | 0 | 0 |
| 2 | Genotoxic carcinogenicity mutagenicity rule | 117 substructures: carcinogenicity or mutagenicity. | 0 | 0 |
| 3 | Nongenotoxic carcinogenicity rule | 23 substructures: carcinogenicity through nongenotoxic mechanisms. | 0 | 0 |
| 4 | Skin sensitization rule | 155 substructures: skin irritation. | 3 alerts | 0 |
| 5 | Acute toxicity rule | 20 substructures: acute toxicity during oral administration. | 0 | 0 |
| 6 | Non-biodegradable | 19 substructures: non-biodegradable. | 1 alert | 0 |
| 7 | SureChEMBL rule | 164 substructures: MedChem unfriendly status. | 0 | 0 |
| 8 | FAF-Drugs4 rule | 154 toxic substructures from FAF-Drugs4 | 1 alert | 1 alert |

**Supplementary Table S3. *In silico* predictions for possible effects of HBP2–3 on Tox21 pathways.**

| **No.** | **Property** | **Comment** | **HBP2** | **HBP3** |
| --- | --- | --- | --- | --- |
| 1 | NR-AhR | Aryl hydrocarbon receptor. The probability of being active.  Inactives: 0; actives: 1. | --  (0.115) | ---  (0.0) |
| 2 | NR-AR | Androgen receptor. The probability of being active.  Inactives: 0; actives: 1. | ---  (0.014) | ---  (0.0) |
| 3 | NR-AR-LBD | Androgen receptor ligand-binding domain.  The probability of being active. Inactives: 0; actives: 1. | ---  (0.011) | ---  (0.0) |
| 4 | NR-Aromatase | The probability of being active. Inactives: 0; actives: 1. | ---  (0.016) | ---  (0.0) |
| 5 | NR-ER | Estrogen receptor.  The probability of being active. Inactives: 0; actives: 1. | --  (0.166) | ---  (0.005) |
| 6 | NR-ER-LBD | Estrogen receptor ligand-binding domain.  The probability of being active. Inactives: 0; actives: 1. | ---  (0.028) | ---  (0.0) |
| 7 | NR-PPAR-gamma | Peroxisome proliferator-activated receptor gamma.  The probability of being active. Inactives: 0; actives: 1. | ---  (0.026) | ---  (0.0) |
| 8 | SR-ARE | Antioxidant response element.  The probability of being active. Inactives: 0; actives: 1. | --  (0.161) | ---  (0.002) |
| 9 | SR-ATAD5 | ATPase family AAA domain-containing protein 5.  The probability of being active. Inactives: 0; actives: 1. | ---  (0.0) | ---  (0.0) |
| 10 | SR-HSE | Heat shock factor response element.  The probability of being active. Inactives: 0; actives: 1. | ---  (0.036) | ---  (0.0) |
| 11 | SR-MMP | Mitochondrial membrane potential.  The probability of being active. Inactives: 0; actives: 1. | ---  (0.096) | ---  (0.0) |
| 12 | SR-p53 | p53, a tumor suppressor protein.  The probability of being active. Inactives: 0; actives: 1. | ---  (0.029) | ---  (0.0) |

For the classification endpoints, the prediction probability values are transformed into six symbols: 0–0.1 (---), 0.1–0.3 (--), 0.3–0.5 (-), 0.5–0.7 (+), 0.7–0.9 (++), and 0.9–1.0 (+++).

**Supplementary Table S4. *In silico* predicted effects of HBP2–3 on the protein levels of caspase-3 and Bcl-2.**

| **I. Upregulation model** | | | | | | | | |
| --- | --- | --- | --- | --- | --- | --- | --- | --- |
| No. | Gene | *IAP* | **HBP2** | | | **HBP3** | | |
|  |  |  | *Pa* | *Pi* | *Pa* > *Pi*, *Pa* ≥ 0.5 | *Pa* | *Pi* | *Pa* > *Pi*, *Pa* ≥ 0.5 |
| 1 | Caspase-3 | 0.887 | 0.725 | 0.009 | Yes | 0.665 | 0.012 | Yes |
| **II. Downregulation model** | | | | | | | | |
| No. | Gene | *IAP* | **HBP2** | | | **HBP3** | | |
|  |  |  | *Pa* | *Pi* | *Pa* > *Pi*, *Pa* ≥ 0.5 | *Pa* | *Pi* | *Pa* > *Pi*, *Pa* ≥ 0.5 |
| 1 | Bcl-2 | 0.892 | 0.632 | 0.014 | Yes | 0.560 | 0.018 | Yes |

*Pa*: probability of being active.

*Pi*: probability of being inactive.

IAP: Invariant Accuracy of Prediction.
